# In Silico, In Vitro, and In Vivo Investigations of Anticancer Properties of a Novel Platinum (II) Complex and Its PLGA Encapsulated Form

**DOI:** 10.1155/bca/2673015

**Published:** 2025-05-25

**Authors:** Zahra Shabaninejad, Mahdiyar Dehshiri, Sayed Mostafa Modarres Mousavi, Maryam Nikkhah, Sadegh Shirian, Sajad Moradi, S. Masoud Nabavizadeh

**Affiliations:** ^1^Department of Nanobiotechnology, Faculty of Biological Sciences, Tarbiat Modares University, P.O. Box 14115-154, Tehran, Iran; ^2^Department of Pathology, Faculty of Veterinary Medicine, Shahrekord University, Shahrekord, Iran; ^3^Shiraz Molecular Pathology Research Center, Dr. Daneshbod Pathology Laboratory, Shiraz, Iran; ^4^Nano Drug Delivery Research Center, Health Technology Institute, Kermanshah University of Medical Sciences, P.O. Box 7616913555, Kermanshah, Iran; ^5^Department of Chemistry, College of Sciences, Shiraz University, P.O. Box 84334-71946, Shiraz, Iran

**Keywords:** antitumor, apoptosis, nanoencapsulation, platinum complex, PLGA

## Abstract

In recent years, the development of multinuclear platinum complexes has introduced a new era in platinum-based chemotherapy, offering improved cytotoxicity and the ability to overcome resistance. However, these complexes still face challenges related to water solubility, biodistribution, and targeted delivery. This study provides a comprehensive investigation of a novel platinum (II) complex, [Pt_2_(μ-bpy-2H) (Me)_2_(dmso)_2_] (C1), focusing on its DNA binding ability and anticancer activity. Computational and experimental approaches revealed that C1 binding to guanine bases and involvement of intercalative interactions. C1 exhibited cytotoxicity in both cisplatin sensitive and resistant cancer cell lines. To enhance the pharmacokinetic and pharmacodynamic properties of C1, it was encapsulated using poly (D, L-lactic-co-glycolic acid) (PLGA). Molecular dynamic simulations predicted the formation of stable C1/PLGA complexes during the early stages of simulation. Encapsulated C1 showed superior antitumor activity with significantly reduced side effects in tumor-bearing mouse models. In conclusion, this study highlights the novel platinum (II) complex C1 as a promising anticancer agent, especially when paired with PLGA encapsulation to improve its effectiveness and reduce side effects.

## 1. Introduction

Since its initial report in 1969 for its antiproliferative activity, cisplatin, or cis-diamminedichloroplatinum (II), has become widely utilized in the treatment of various cancers [1]. Within cells, cisplatin undergoes hydrolysis and substitution reactions, leading to the loss of one or both chloro ligands. The hydrolyzed form then reacts with sulfhydryl groups in proteins and nucleic acids, forming a closed ring and bis-adducts with DNA [2]. Despite its numerous advantages, the clinical use of cisplatin has been hindered by significant drawbacks, including intrinsic resistance in certain cancer cells and a broad range of side effects [3]. Consequently, extensive efforts have been directed toward the development of novel platinum-based complexes with enhanced therapeutic potential and reduced toxicity in normal cells [4, 5].

Multiplatinum complexes have demonstrated the ability to overcome drug resistance in certain human cancer cells and to form extended DNA adducts that differ significantly from those created by cisplatin. The initial report of therapeutic anticancer properties of a di-nuclear platinum complex marked the beginning of a transformative era in platinum-based chemotherapy [6]. This groundbreaking discovery led to a paradigm shift. In 1989, Farrell et al. introduced an innovative di-nuclear complex consisting of two cisplatin platinum centers connected by a variable aliphatic diamine chain [7]. They subsequently expanded the design to include complexes with both cis- and trans-platinum centers, and replaced the chloro ligands with malonate to improve water solubility [8–10]. Around the same time, Broomhead et al. synthesized a comparable series of di-nuclear platinum complexes [11, 12], but their complexes were linked with the 4,4′-dipyrazolylmethane ligand. Recently, several multinuclear platinum complexes have advanced to clinical trials, yielding a range of outcomes [6, 13]. This group of charged complexes, including di- and tri-nuclear compounds connected by aliphatic ligands, has demonstrated the ability to overcome resistance to cisplatin and carboplatin in several significant human cancer cell lines [14, 15]. Multinuclear platinum DNA adducts are distinguished by their flexibility, nondirectionality, and propensity for forming interstrand cross-links. Their increased cytotoxicity is largely attributed to the formation of flexible, non-directional DNA adducts, predominantly interstrand cross-links. Additionally, these complexes can induce conformational changes in DNA, particularly transitions from B-type to Z-type and A-type structures [6, 16, 17].

The use of nanomaterials for delivering platinum complexes has emerged as a promising strategy to mitigate their side effects and resistance in cancer cells. These nanomaterials include carbon dots [18], carbon nanotubes [19], graphene [20], silica nanoparticles [21], iron oxide nanoparticles [22], gold nanomaterials [23], upconversion nanoparticles [24], polymeric nanoparticles [25], liposomes [26], vesicles [27], polymeric micelles [28] and dendrimers [29, 30]. Poly(lactic-co-glycolic acid) (PLGA) as a biocompatible, biodegradable, and FDA-approved polymer has been extensively utilized in [31–34] the formulation of polymeric nanosystems. Its widespread application is attributed to several key properties, including biocompatibility, biodegradability, the ability to facilitate sustained release, feasible surface modifications. Additionally, PLGA offers protection against drug degradation, well-established preparation and synthesis techniques suitable for various drug types (e.g., hydrophilic or hydrophobic small molecules and macromolecules), and the capacity to target specific organs or cells [35].

**Figure 1:**
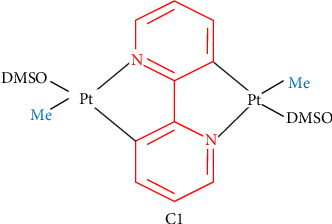
Molecular structure of C1. Me is deprotonated methyl and DMSO is deprotonated dimethyl sulfoxide.

In the present study, the DNA-binding ability and cytotoxicity of a novel platinum complex, [Pt_2_(μ-bpy-2H) (Me)_2_(dmso)_2_], referred to as C1, were investigated across different cell lines. As shown in Figure 1, two nuclear platinum centers are connected via a 2,2′-bipyridine (bpy) group. The two reactive platinum centers, along with the bpy group, are expected to enhance the complex DNA-binding affinity and lipophilicity, respectively. The presence of two platinum centers may cause distinct DNA lesions, contributing to increased toxicity. Additionally, the DMSO groups may facilitate the transmembrane transport of the complex across the plasma and nuclear membranes [36]. To enhance the C1 antitumor activity, by passive targeting mechanisms, it was encapsulated in PLGA nanoparticles, and its cytotoxicity was compared in cisplatin sensitive and resistant cell lines. The capability of the nanoencapsulated C1 in tumor growth inhibition was compared with nanoencapsulated cisplatin in murine breast cancer model.

## 2. Materials and Methods

### 2.1. Materials

Bovine spleen DNA, 3-(4,5-Dimethylthiazol-2-yl)-2,5-diphenyltetrazolium bromide (MTT), trypan blue and poly vinyl alcohol (PVA) (14 KDa) were purchased from Sigma Co., Germany. Ethidium bromide was obtained from Cinnagen Co., Iran. Cisplatin (1 mg/mL) was provided by Mylan, France. PLGA (180 KDa) was purchased from Alborz Nanomed Tech Company. All other materials and reagents were obtained from Merck Millipore Co. and were of analytical grade.

### 2.2. Methods

#### 2.2.1. C1 Synthesis

[Pt_2_(μ-bpy-2H) (Me)_2_(dmso)_2_], referred to as C1 was synthetized according to a pervious report [37]. The initial complex [Pt_2_(μ-bpy-2H) (Me)_2_(dmso)_2_] was synthesized through the reaction of [Pt(Me)_2_(dmso)_2_] with 0.5 equivalents of 2,2′-bpy in dry toluene under reflux condition at 110°C and argon atmosphere. In this complex, bpy-2H refers to a double deprotonated 2,2′-bpy ligand, while dmso stands for dimethylsulfoxide.

#### 2.2.2. Spectroscopic Analysis of C1 Binding to DNA

Interaction of C1 with DNA was studied in TN buffer (Tris buffer (5 mM), pH 7.2 and NaCl (50 mM)) by UV-visible spectroscopy (T 90^+^ Spectrophotometer, England). The absorption spectra of C1 were recorded 5 min after the addition of increasing concentrations of DNA (0–2.6 μM). The competitive binding of C1 and ethidium bromide (10 μM) to DNA (100 μM) was further analyzed by ethidium bromide fluorescence measurements in the presence of increasing amount of C1. The emission spectra of the mixture were recorded at 550–670 nm, at the excitation wavelength of 546 nm using a Cary-Eclipse spectrofluorometer (Model Varian, Australia).

#### 2.2.3. Molecular Docking and In Silico Investigations of C1 Binding to DNA

The atomistic details of molecular interactions between C1 and DNA, the main target of platinum-based anticancer drugs, were investigated using molecular docking. Docking studies were conducted with the freeware AutoDock 4 [38]. Nonpolar hydrogens were merged, atomic charges were added, and AutoDock atom typing was performed using the MGLTools package. All bonds were considered active, and the search space was set large enough to ensure DNA's accessibility for all potential ligand binding sites. Energetic maps were calculated for each atom type on the grid points, separated by a distance of 0.375 Å, using AutoGrid4 software. Finally, a maximum of 200 docking runs was performed utilizing the Lamarckian genetic algorithm.

#### 2.2.4. MTT Assay

MCF7 (breast adenocarcinoma) was cultured in HDEMEM (Gibco). A2780 (ovarian cancer cell line), A2780R (ovarian cancer cell line resistant to cisplatin), and MRC5 (fibroblast lung tissue) cell lines were cultured in RPMI-1640 (Gibco). The culture media were supplemented with 10% fetal bovine serum (Gibco), 100 μg/mL streptomycin (sigma), and 100 U/mL penicillin. The cells were incubated in a standard cell culture incubator. All the cell lines were obtained from the Iranian Biological Resource Center.

After reaching the confluency of 70%–80%, the cells were separately treated with C1, cisplatin and their PLGA encapsulated forms at different concentrations in 96-well plates for 24 h. Then 10 μL of MTT stock solution (5 mg/mL) was added to each well and the cells were incubated at 37°C for 3 h. The cell culture media were aspirated, and the purple formazan crystals were dissolved by 100 μL of DMSO. Absorbance was measured at 570 nm using a microplate reader (Bio-Tek ELx808, USA).

#### 2.2.5. Annexin V-PI Staining of the C1-Treated Cells

The cells were treated with cisplatin and C1 for 12 h. The treated cells were trypsinized and resuspended in 500 μL of binding buffer. Then, 2 μL of AnnexinV-FITC and 2 μL of propidium iodide (PI) were added to the cells and incubated at room temperature in dark for 20 min. The stained cells were analyzed with a flow cytometer (BD Bioscience instrument, USA).

#### 2.2.6. Extraction of Total Protein and Genomic DNA (gDNA) From C1-Treated Cells

Cells cultured in T75 flasks were treated individually with C1, cisplatin, and their PLGA-encapsulated forms for 24 h. Subsequently, the cells were trypsinized and divided equally into three tubes for protein extraction, DNA extraction, and cell uptake measurements. The cells were harvested via centrifugation at 160 × g for 5 min. One portion, designated for cell uptake assessment, was stored at −20°C until platinum concentration measurements (details below). The second portion was used for protein extraction, where cell pellets were washed twice with ice-cold phosphate-buffered saline (PBS). Then, 1 mL of ice-cold RIPA buffer (containing Tris-HCl [50 mM], pH 7.4; NaCl [150 mM]; EDTA [1 mM]; NP-40 [1%]; sodium deoxycholate [1%]; and SDS [1%]), supplemented with protease inhibitor cocktail, was added to the cells. After incubation at −20°C for 20 min, the mixture was centrifuged at 16, 000 × g for 20 min at 4°C. The supernatant was collected, and the protein concentration was quantified using a BCA kit (Thermo Fisher Company) according to the manufacturer's instructions.

The third portion of cells was used for gDNA purification using the PureLink gDNA Kit (Invitrogen). Briefly, the harvested cells were resuspended in 200 μL of PBS and treated with proteinase K and RNase A. After adding 200 μL of binding buffer, the cells were incubated at 55°C for 10 min, followed by the addition of 200 μL of absolute ethanol. The resulting lysates were loaded onto spin columns and centrifuged. The columns were then washed with washing buffer, and the gDNA was eluted using 200 μL of elution buffer.

#### 2.2.7. Platinum Concentration Measurements by Inductively Coupled Plasma-Optical Emission Spectrometry (ICP-OES)

The platinum content in the harvested cells and their extracted gDNA was measured using ICP-OES. The samples were digested overnight at room temperature in a 4:1 mixture of HNO_3_ (200 μL) and H_2_O_2_ (50 μL) followed by addition of 650 μL of HNO_3_ [39]. The platinum concentrations were quantified using a Varian 730-ES ICP-OES instrument. The measured platinum content in the cells and gDNA for each sample was normalized to the total protein concentration and DNA concentration, respectively.

#### 2.2.8. Molecular Dynamics Simulation of C1/PLGA Nanoparticles Formation

The molecular structures of C1 and PLGA were initially sketched in two dimensions and subsequently optimized to three-dimensional structures using Gaussian 9 software [40]. The Hartree–Fock method was employed utilizing the basis sets LANL2DZ and 6–311G for C1 and PLGA, respectively. The topological information for both molecules was determined using Antechamber software. [41]. A 200 ns MD simulation was conducted using the GROMACS molecular package (Version 2021) [42] and amber-eilden-99sb force field. The same drug-to-carrier ratio used in experimental studies (4PLGA:15MOL) was randomly dispersed within the simulation box, which was then filled with the TIP3P water model and neutralized by the addition of appropriate counter ions. Inter- and intra-atomic interactions were relaxed using the steepest descent algorithm. Temperature and pressure were set to 300 K and 1 bar, respectively, utilizing NVT and NPT ensembles. *n* this regard, the Nosé-Hoover thermostat [43] and the Parrinello-Rahman barostat [44] were applied. Short-range Coulombic and Van der Waals interactions were computed using a calculation cut-off of 1.2 nm. Long-range electrostatic forces were evaluated using the Particle Mesh Ewald method [45]. The production run was carried out for 200 ns using the leapfrog algorithm [46]. Three-dimensional representations of molecular interactions were generated using the MGLTools and VMD packages [47].

#### 2.2.9. Encapsulation of C1 by PLGA

C1 and cisplatin were loaded onto PLGA nanoparticles using the emulsion-evaporation method. Briefly, 1 mL of cisplatin (1 mg/mL), or 1 mL of C1 (2, 4, 6, 8 mg/mL) in DMSO were added to 1 mL of PLGA (15 mg/mL) in dichloromethane. The mixture was added drop wise to 20 mL of PVA (1%) under vigorous stirring. The suspension was sonicated for 2 min (60 mV) and then the organic phase was evaporated by stirring the suspension for 3–5 h at room temperature. The nanoparticles were collected by centrifugation at 12, 000 × g for 20 min. The pellets were resuspended in appropriate volumes of Milli Q water and sonicated for 2 min (60 mV). The size and zeta potential of nanoparticles were determined by a Malvern Zetasizer Nano ZS instrument with DTS software (Malvern Instruments, UK). The morphology of nanoparticles was analyzed by a scanning electron microscopy (MIRA3 TESCAN, Czech Republic).

#### 2.2.10. In Vivo Studies

BALB/c mice bearing tumors, developed by subcutaneous injection of 4T1 cells at dorsal flank, were purchased from the Institute of Biochemistry and Biophysics, Tehran University. The mice were housed under controlled conditions (ambient temperature 22 ± 2°C, 12 h light/dark cycle) with food and water ad libitum. All experiments were carried out according to the protocol approved by biomedical research ethics committee of Tarbiat Modares University. The mice were randomly divided into three groups (*n* = 4) including control, treated by encapsulated C1 (C1-NP) and encapsulated cisplatin (cisplatin-NP) intravenously. Treatments (1 mg/kg, IV) were administered once a week for 1 month (4 doses) and the animals were monitored daily. Tumor volume was calculated by modified ellipsoidal formula: Tumor volume = ½ (Length × weight 2).

One week after injection of the fourth dose, mice were deeply anesthetized by intraperitoneal injection of ketamine and xylazine followed by cervical dislocation to assure euthanasia. Then, organs removal was done for further analysis. Lung, spleen, liver, and kidney of each mouse were removed for macroscopic and microscopic evaluation. The organs were immediately fixed in 10% neutral buffered formalin. The samples were then dehydrated in graded ethanol and embedded in paraffin. Sections of 5 μm thickness were stained with hematoxylin and eosin and examined by light microscopy.

#### 2.2.11. Statistical Analysis

Experimental results were presented as the mean ± standard deviation (SD), analyzed using GraphPad PRISM 8 software (GraphPad Software). Statistical significance was determined using two-tailed Student's *t*-tests or two-way ANOVA and repeated measures ANOVA. A *p* value of less than 0.05 was considered statistically significant.

## 3. Results and Discussion

### 3.1. Evaluation of C1 Binding to DNA

#### 3.1.1. UV-Visible Absorption Spectroscopy

**Figure 2:**
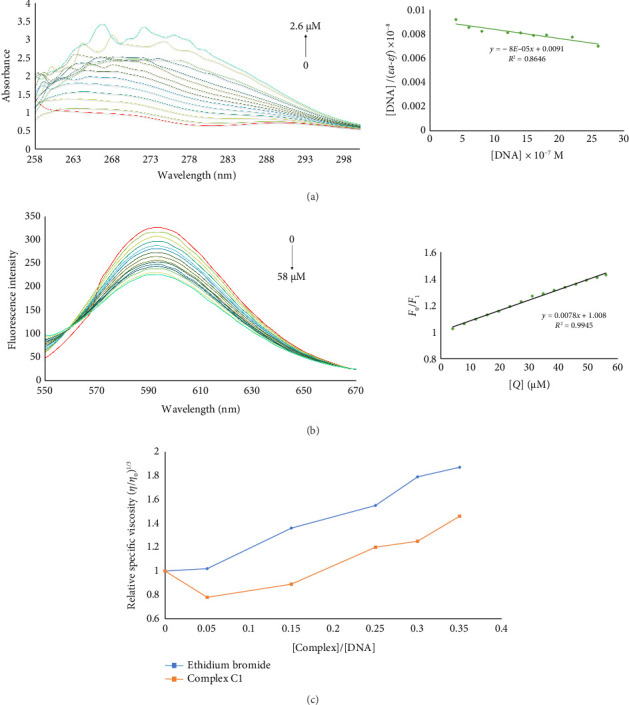
(a) The absorption spectra of C1 (50 μM) in the presence of increasing concentration of DNA (0–2.6 mM). The red spectrum is the absorption of C1 in the absence of DNA. The inset is [DNA]/(*ε*_*a*_-*ε*_*f*_) versus [DNA] plot. (b) Emission spectra of ethidium bromide (100 μM) mixed with DNA (10 μM) in the presence of increasing concentration of C1 (0–58 μM). The emission spectrum of ethidium bromide in the absence of C1 is shown in red. The inset indicates the *F*_0_/*F*_1_ versus [*Q*] or [C1] plot. (c) Relative viscosity changes of a DNA solution upon addition of increasing amounts of ethidium bromide and C1.

The interaction of metal complexes with DNA may cause remarkable alternation in their optical properties including maximum absorbance wavelength and/or absorbance intensity [48, 49]. The interactions such as the coordination of the nitrogenous base of DNA with the metal center of complexes, intercalation, and electrostatic interactions between the metal center and phosphate groups of DNA are usually involved in metal complexes binding to DNA [50, 51]. As shown in Figure 2(a), increasing the concentration of DNA (0–2.6 μM) caused a hyperchromic effect in UV-visible spectra of the C1. This observation suggests that the C1 can be a groove binder [52, 53]. The following equation was used to calculate the binding constant (*K*_*b*_):

(1) $$\begin{array}{c} \frac{\left[DNA\right]}{\varepsilon_{a}-\varepsilon_{f}}=\frac{\left[DNA\right]}{\varepsilon_{b}-\varepsilon_{f}}+\frac{1}{K_{b}\left(\varepsilon_{b}-\varepsilon_{f}\right)}, \end{array}$$

where [DNA] represents the concentration of DNA, *ε*_*a*_ denotes the absorbance/[C1] ratio, *ε*_*f*_ is the extinction coefficient of free C1 and *ε*_*b*_ is the extinction coefficient of C1 in its fully bound form with DNA. The *K*_*b*_ is the ratio of slope to intercept from the plot of [DNA]/(*ε*_*a*_-*ε*_*f*_) versus [DNA] [43]. The calculated *K*_*b*_ value of 8.8 × 10^4^ M^−1^ indicates a moderate affinity of C1 for DNA.

#### 3.1.2. The Fluorescence Spectroscopy

Ethidium bromide is a well-known intercalative fluorescent dye that exhibits a 20–25 fold enhancement in fluorescent emission intensity upon intercalation with DNA The displacement of ethidium bromide by intercalative agent results in the quenching of its fluorescence intensity [54]. A reduction in ethidium bromide fluorescence was observed with increasing concentrations of C1 (0–58 μM). The Stern–Volmer plot is presented in Figure 2(b). The Stern–Volmer equation is as follows:

(2) $$\begin{array}{c} \frac{F_{0}}{F_{1}}=1+K_{SV}\left[Q\right], \end{array}$$

where *K*_*SV*_ represents the Stern–Volmer quenching constant, [*Q*] is the molar concentration of the quencher, and *F*_0_ and *F*_1_ are fluorescence intensities of the ethidium Bromide in the absence and presence of the quencher, respectively [55]. The calculated *K*_*SV*_ for C1 was 7.8 × 10^3^ M^−1^. This value is comparable to those of previously reported platinum-containing compounds known to intercalate with DNA [56, 57].

#### 3.1.3. Viscosity Measurement

To further explore the binding mode of C1, viscosity changes in a DNA solution were examined in the presence of increasing concentrations of ethidium bromide and the C1. The intercalation of small molecules into DNA leads to an increase in the axial length of DNA, resulting in a higher frictional coefficient and increased viscosity of the DNA solution [58, 59]. As shown in Figure 2(c), the relative viscosity of the DNA solution rose with increasing concentrations of C1, suggesting that C1 engages in intercalative interactions with DNA.

#### 3.1.4. Analysis of the C1/DNA Binding by Molecular Docking

**Figure 3:**
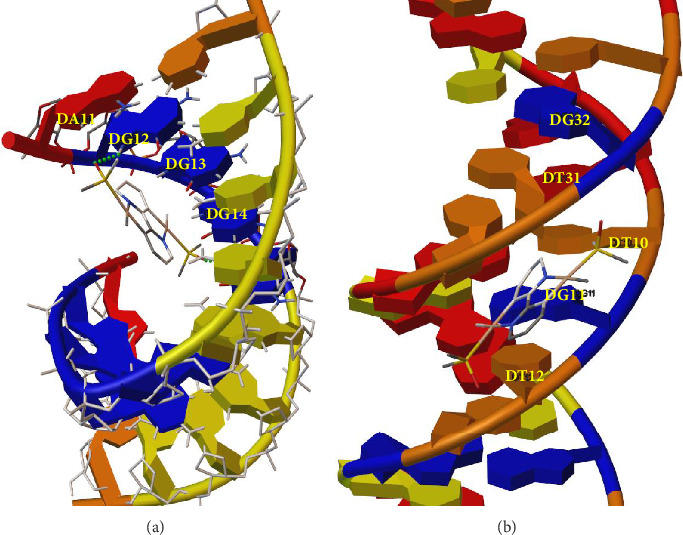
Molecular interactions of C1 with (a) a poly-G DNA and (b) a random sequence of DNA.

Molecular docking was employed to analyze the atomic interactions between C1 and DNA. A random sequence of B-DNA and a poly-G sequence were evaluated as molecular targets for C1. The results confirmed the formation of stable complexes between C1 and both DNA sequences (Figure 3). In both cases, C1 interacted with guanine bases, which serve as the primary targets for platinum coordination. As previously reported, platinum coordination occurs via the N7 atoms of two adjacent guanine bases in poly-G DNA. Additionally, the stable complex formation of C1 within the minor groove of DNA in nonpoly-G sequences may potentially block polymerization and/or repair mechanisms in cancer cells.

### 3.2. Cytotoxicity Assessment of C1 Compared to Cisplatin

#### 3.2.1. MTT Assay of Cells Treated With C1 and Cisplatin

**Figure 4:**
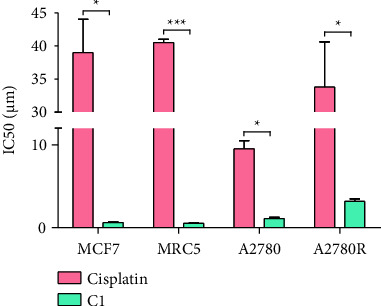
The IC50 values (μM) of cisplatin and C1, determined by treatment of MCF7, MRC5, A2780, and A2780R cell lines for 24 h. The statistical analysis was performed with a two-tailed student's *t*-test (*p* value < 0.05). ^∗^indicates *p* value < 0.05 and ^∗∗∗^indicates *p* value < 0.001.

The cytotoxicity of C1 was compared to cisplatin in different cell lines including MCF7, MRC5, A2780, and A2780R cell lines. C1 demonstrated high antiproliferative activity in the tested cells and was significantly more cytotoxic than cisplatin (Figure 4 and Figure S1). Generally, complexes containing more than one platinum (II) center exhibit greater cytotoxic effects compared to single-nuclear complexes [60, 61]. However, the antitumor properties of these complexes are influenced by multiple factors, including the extent and mode of cell membrane penetration, cellular accumulation and distribution, DNA interaction levels, sensitivity to detoxification mechanisms, cell type characteristics, and the efficiency of DNA repair mechanisms [62, 63].

#### 3.2.2. Annexin V-PI Staining of the Treated Cells With C1 and Cisplatin

**Figure 5:**
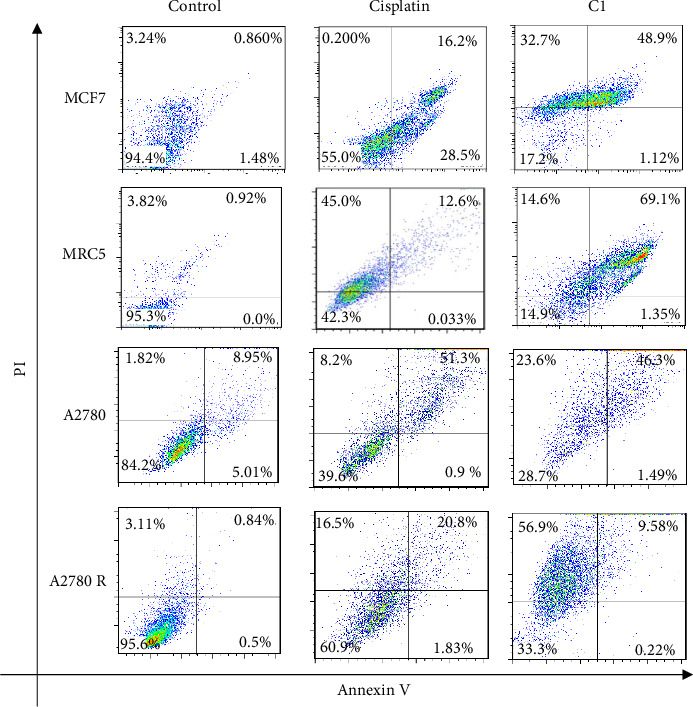
The annexin V-PI staining of MCF7, MRC5, A2780 and A2780R cells treated with cisplatin and C1 for 24 h. Viable cells, early apoptotic, late apoptotic, and nonviable necrotic cells appear in the lower left, lower right, upper right and upper left quadrants, respectively.

Apoptosis induction in cells treated with C1 and cisplatin was analyzed using Annexin V-PI staining and flow cytometry (Figure 5). The findings revealed that C1 exhibited higher cytotoxicity compared to cisplatin across all the studied cancer cell lines. However, C1 has high toxicity towards MRC5 cells that are fetal cells derived from normal human lung tissue. The toxicity of C1 on normal cells was expected as the chemotherapeutic compounds often affect normal cells. Currently, the prolonged use of cisplatin and cisplatin-derivatives is hampered by off-target toxicity leading to the occurrence of major side effects in a significant percentage of patients. Acute kidney injury, gastrointestinal disorders, hemorrhage, ototoxicity, neurotoxicity and suppressed immune system are among the most common side-effects observed [64]. Targeted drug delivery to cancer cells significantly minimizes off-target toxicity of cytotoxic drugs.

#### 3.2.3. Cellular Uptake and DNA Binding Capacity of C1

**Figure 6:**
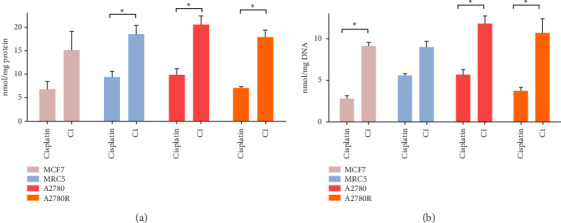
Platinum content of (a) the cells treated with cisplatin and C1, (b) the extracted gDNA from the cells treated by cisplatin and C1. The data were normalized to the total protein and DNA concentrations, respectively. The statistical analysis was performed with a two-tailed student's *t*-test (*p* value < 0.05). ^∗^indicates *p* value < 0.05.

The cellular uptake and gDNA binding efficiency of C1 and cisplatin were compared in MCF7, MRC5, A2780, and A2780R cell lines by measuring the platinum content in the cells (total) and the extracted gDNA (Figure 6). The DNA binding of C1 was proportional to its rate of cellular uptake. Overall, the cell accumulation and DNA binding of C1 appeared to be higher than those of cisplatin. This observation may be attributed to the hydrophobic nature of C1.

### 3.3. Encapsulation in PLGA and Investigation of Biological Effects of Encapsulated Forms of C1 and Cisplatin

#### 3.3.1. Molecular Dynamic Simulation of C1/PLGA Complex Formation

**Figure 7:**
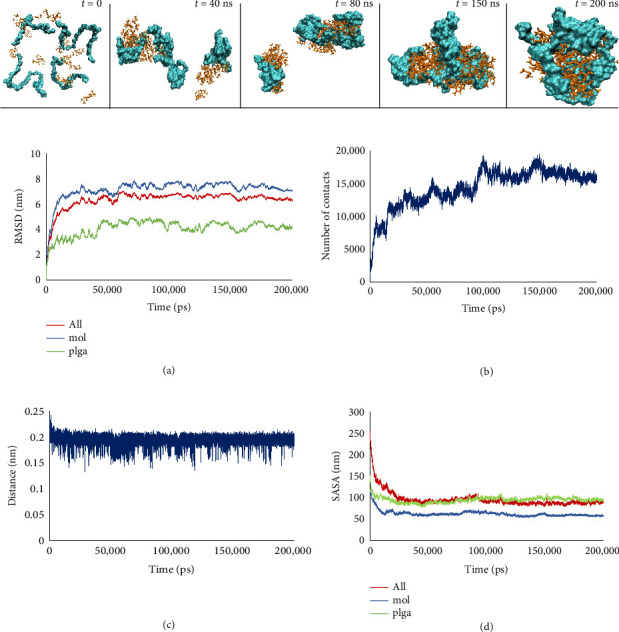
Snapshots of the simulation of C1/PLGA complex formation (top), analysis of (a) RMSD, (b) number of contacts, (c) the intermolecular distance and (d) solvent accessible surface area of the system.

The complex formation between PLGA and C1 was analyzed using molecular dynamics simulations. As shown in Figure 7, primary complexes were observed during the early stages of the simulation, indicating a high affinity of C1 for forming molecular complexes with PLGA. The plateau observed in the RMSD diagram (Figure 7(a)) confirms the stable complex formation between the molecules. Consistent with the RMSD results, the analysis of the number of contacts (Figure 7(b)) and changes in intermolecular distances (Figure 7(c)) during the simulation further supports the robust establishment of molecular complexes in the system. Additionally, the results of the solvent-accessible surface area (SASA) analysis (Figure 7(d)) reveal that the drug is located in the inner regions of the nanoparticles and is encapsulated by PLGA.

#### 3.3.2. Encapsulation of C1 and Cisplatin by PLGA

Encapsulation of therapeutic compounds within polymers has been recognized as a promising strategy to reduce toxicity toward normal cells [65]. Polymer-based drug delivery systems contribute to drug protection and minimize exposure to normal cells, thereby reducing off-target effects. In tumor environments characterized by leaky vasculature and an impaired lymphatic system, polymer-based systems can deliver cargo to cancer cells through the enhanced permeability and retention (EPR) effect, resulting in the accumulation of the compound in tumor tissues. [66, 67]. PLGA is a biodegradable and biocompatible FDA-approved polymer which is widely used in therapeutic applications [68]. To enhance efficiency and minimize off-target effects, C1 was encapsulated in PLGA. Various concentrations of the C1 were utilized during the encapsulation process to achieve optimal encapsulation efficiency. Cisplatin was also encapsulated and served as a control. The encapsulation efficiency was assessed using ICP-OES and calculated based on the following equation:

(3) $$\begin{array}{c} \text{Encapsulation Efficiency}\left(\%\right)=\left(\frac{W_{i}}{W_{t}}\right)\times 100, \end{array}$$

where the *W*_*i*_ represents the weight of the encapsulated compound and *W*_*t*_ is the total weight of the compound used in the synthesis reaction [69]. The results are summarized in Table 1. The synthesized nanoparticles were approximately 200–300 nm in size and carried a negative charge. The highest encapsulation efficiency was observed with 8 mg/mL of C1 in the encapsulation reaction. However, higher concentrations of C1 led to the formation of large aggregates.

**Figure 8:**
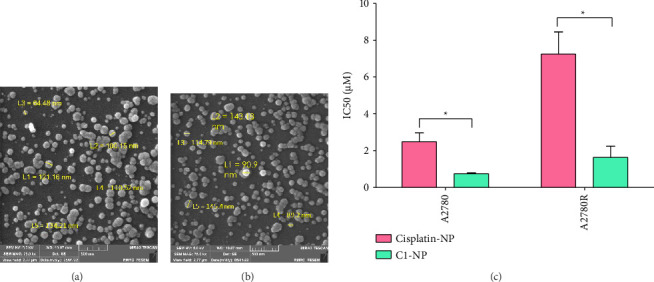
SEM images of (a) cisplatin-NP, (b) C1-NP. The scale bar is 500 nm (c) IC50 values (μM) of cisplatin-NP and C1-NP, determined by treatment of A2780, and A2780R cell lines for 24 h. The statistical analysis was performed using two-tailed student's *t*-test (*p* value < 0.05). ^∗^indicates *p* value < 0.05.

SEM imaging of cisplatin-NP and C1-NP showed their spherical morphology, with average sizes of 110.52 ± 23.68 nm and 114.79 ± 24.29 nm, respectively (Figures 8(a) and 8(b)).

#### 3.3.3. Evaluation of C1-NP and Cisplatin-NP Cytotoxicity

##### 3.3.3.1. Cytotoxic Effects of C1-NP and Cisplatin-NP on A2780 and A2780R Cells

A2780 (sensitive to cisplatin) and A2780R (resistant to cisplatin) cell lines were treated with both the encapsulated and free form of cisplatin and C1 at various concentrations (Figure 8(c) and Figure S2). Based on encapsulation efficiency, the quantities of C1-NP and cisplatin-NP were calculated to deliver 0–400 μM of the encapsulated compounds. Both encapsulated forms demonstrated higher cytotoxicity compared to their free counterparts, highlighting the enhanced biological activity of C1 and cisplatin achieved through PLGA encapsulation. C1-NP exhibited superior antiproliferative effects. Notably, the IC50 values of C1 and C1-NP were approximately 10- and 6-fold lower than those of cisplatin and cisplatin-NP, respectively.

##### 3.3.3.2. Antitumor Effects of Cisplatin-NP and C1-NP in Mice Cancer Models

**Figure 9:**
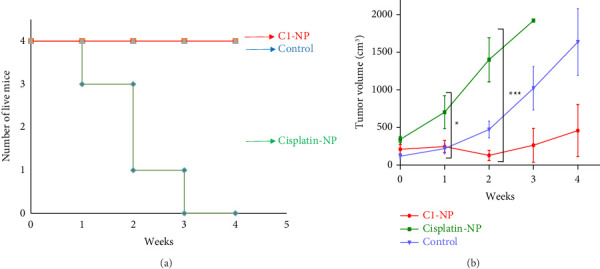
(a) Survival plot and (b) tumor volumes of control, cisplatin-NP, and C1-NP treated mice. Treatments were done through the tail vein once a week. The statistical analysis was performed using two-way ANOVA and repeated measures ANOVA (*p* value < 0.05). ^∗^indicates *p* value < 0.05 and ^∗∗∗^indicates *p* value < 0.001.

C1-NP and cisplatin-NP, at a dosage equivalent to 1 mg/kg, were administered intravenously into the tail vein of tumor-bearing mice. The injections were performed four times at 1 week intervals.  Figure 9(a) illustrates the survival rates of mice during the treatment, while Figure 9(b) depicts the tumor volumes measured after each injection. In the cisplatin-NP-treated group, 1 mouse was lost in the first week, two in the second week, and one in the third week, potentially due to cisplatin-related side effects. The organs were promptly dissected following death and preserved in formalin for histological analysis. No mice were lost in control or C1-NP-treated groups (Figure 9(a)). Consistent with the in vitro findings, the results in mouse models further confirmed the remarkable tumor-suppressive efficacy of the C1-NP complex (Figure 9(b)).

**Figure 10:**
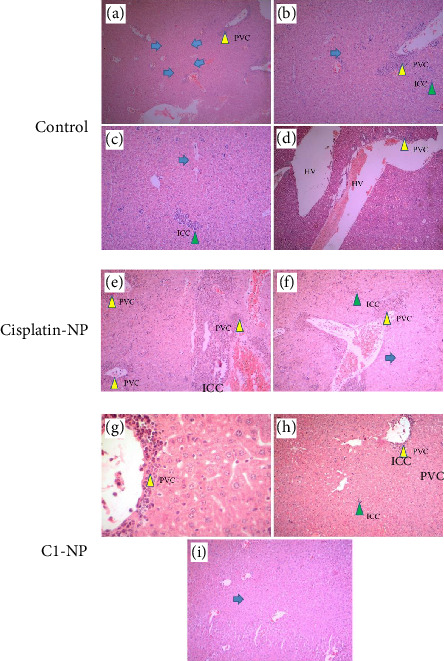
Histological changes of liver tissues of mice treated with cisplatin-NP (e, f) and C1-NP (g–i) compared to controls (a–d). Please refer to the text for definitions of arrows, arrowheads, and color codes.

**Figure 11:**
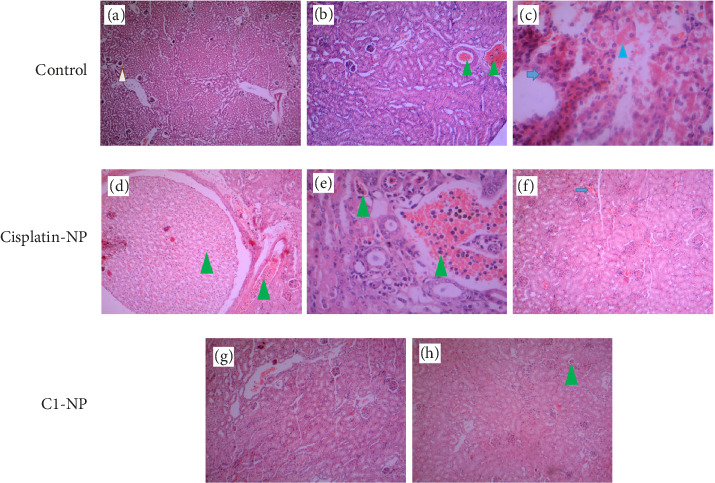
Histological changes of kidney tissues of mice treated with cisplatin-NP (d–f) and C1-NP (g, h) compared to controls (a–c). Please refer to the text for definitions of arrows, arrowheads, and color codes.

**Figure 12:**
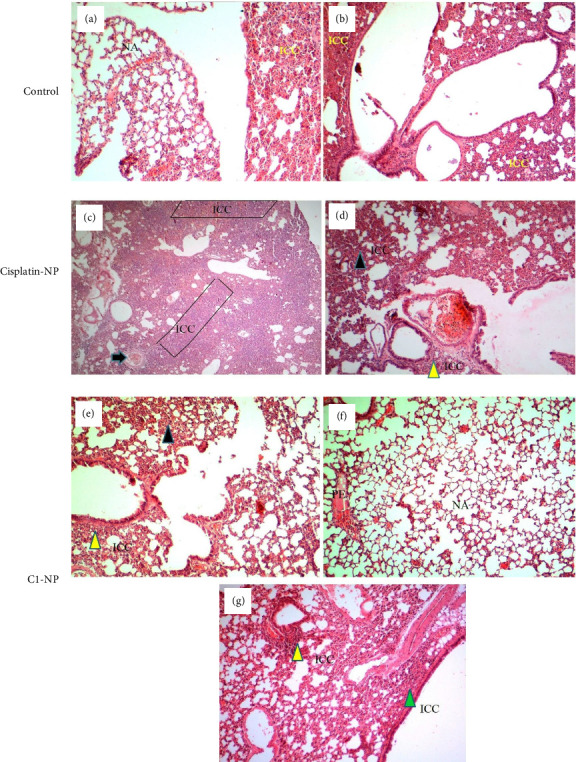
Histological changes of lung tissues of mice treated with cisplatin-NP (c, d) and C1-NP (e–g) compared to controls (a, b). Please refer to the text for definitions of arrows, arrowheads, and color codes.

In Figure S3 morphology and size of various organs of the C1-NP- and cisplatin-NP-treated mice were compared to control (receiving no treatment). Phenotypic abnormalities, including splenomegaly were clearly observed in cisplatin-NP-treated mice. Splenomegaly may result from liver inflammation or viral infections, and this complication has been previously reported in some patients undergoing cisplatin treatment [70, 71]. In C1-NP treated group the organs were more similar to control and the tumors disappeared completely in two of the C1-NP mice in the group (Figure S3). The H&E stained tissues of liver, kidney and lung are presented in Figures 10, 11, 12, respectively. The liver tissue in the control group exhibited a disrupted histological structure, characterized by moderate to severe infiltration of cancer cells (ICC) around the central vein and periportal vessels (perivascular cuffing (PVC) marked by yellow arrowheads). This was accompanied by moderate to severe necrosis (blue arrows) and hypertrophic vessels (HV), as shown in Figures 10(a), 10(b), 10(c), 10(d). In the cisplatin-NP-treated group, the liver displayed significant abnormal histological characteristics, marked by extensive ICC within the liver parenchyma (indicated by green arrowheads) and surrounding the central vein and other blood vessels (highlighted by yellow arrowheads) (Figures 10(e) and 10(f)). Additionally, PVC (yellow arrowhead) and moderate necrosis (blue arrows) were observed. In contrast, the liver tissues of the C1-NP-treated group mostly displayed normal histological structures, with ICC and PVC rarely observed near the central vein (Figures 10(g), 10(h), 10(i)). The kidney in the control group showed signs of histological damage, including mild ICC within vessels, mild to moderate acute tubular necrosis (blue arrowheads), glomerular atrophy (white arrowheads), and the presence of anaplastic cells (blue arrow) (Figures 11(a), 11(b), 11(c)). In the cisplatin-NP-treated group, congestion in extraglomerular vessels along with ICC (green arrowheads) and acute tubular necrosis were evident (Figures 11(d), 11(e), 11(f)). However, the kidney tissues of the C1-NP-treated group primarily displayed normal histological features, with rare ICC in extraglomerular vessels (Figures 11(g) and 11(h)). Normal alveolar (NA) structures and moderate to severe ICC within lung parenchyma were observed in the lung tissues of control group) (Figures 12(a) and 12(b)). The cisplatin-NP-treated group showed severe ICC in the lung parenchyma (box), bronchioles (yellow arrowhead), alveolar walls (black arrowhead), and arterioles (black arrow) (Figures 12(c) and 12(d)). The lung tissues of the C1-NP-treated group predominantly exhibited normal histological structures, although some ICC was noted around the bronchi (green arrowhead), bronchioles (yellow arrowhead), and alveolar walls (black arrowhead), alongside mild pulmonary edema (Figures 12(e), 12(f), 12(g)).

## 4. Conclusion

C1 features a distinctive molecular structure with two nuclear platinum centers linked by a 2,2′-bpy group, which may enhance its DNA binding capacity. It is proposed that C1 interacts with DNA via intercalation. The compound displayed exceptional cytotoxicity in both cisplatin-sensitive and cisplatin-resistant cell lines. Notably, it demonstrated pronounced efficacy against A2780R cells, a cisplatin-resistant cell line. To address issues related to drug solubility, biodistribution, and targeted delivery, C1 was encapsulated within PLGA nanoparticles. This encapsulation not only increased its cytotoxicity compared to free C1 but also highlighted the potential of nanocarrier-based systems to improve the therapeutic efficiency of platinum-based complexes. In vivo studies using tumor-bearing mouse models further validated the significant tumor growth suppression achieved with C1-NPs. C1 ability to overcome cisplatin resistance and its compatibility with nanocarrier-based drug delivery systems present a promising opportunity for advancing platinum-based chemotherapy. Nevertheless, further in vivo studies are essential to evaluate the general toxicity of C1 and to assess its safety and efficacy in cancer treatment. These findings provide a foundation for investigating C1 as a promising candidate for cancer therapy, especially when combined with targeted nanocarriers to minimize toxicity to normal cells.

## Figures and Tables

**Table 1:** Size and zeta potential of C1-NP and cisplatin-NP.

Nanocarriers	Concentration of C1 (mg/mL)	Size (nm)	*ς* potential (mV)	Encapsulation efficiency (%)
C1-NP	2	220 ± 23.6	−21 ± 7.16	42.61 ± 7.68
	4	210 ± 16.35	−19 ± 6.84	49.3 ± 5.62
	6	248 ± 50.19	−23 ± 8.3	58.06 ± 12.56
	8	262 ± 32.81	−20 ± 7.75	63.37 ± 13.67

Cisplatin-NP	1	174.5 ± 14.35	−22.4 ± 8.3	56.13 ± 8.79

## Data Availability

The data that support the findings of this study are available on request from the corresponding author.

## Supporting Information

Supporting Information is available from the Wiley Online Library or from the author. Figure S1: Viability of MCF7, MRC5, A2780, and A2780R cells after treatment with different concentrations of cisplatin and C1. The cytotoxicity was measured by MTT assay after 24 h (green) and 48 h (purple) of treatments. Figure S2: Cell viability of A2780 and A2780R cells after 24 h (green) and 48 h (purple) treatment by cisplatin-NP and C1-NP. Figure S3: Morphology of organs in mice treated with C1-NP and cisplatin-NP compared to control.

## References

[1] Dasari S., Bernard Tchounwou P. (2014). Cisplatin in Cancer Therapy: Molecular Mechanisms of Action. *European Journal of Pharmacology*.

[2] Tchounwou P. B., Dasari S., Noubissi F. K., Ray P., Kumar S. (2021). Advances in Our Understanding of the Molecular Mechanisms of Action of Cisplatin in Cancer Therapy. *Journal of Experimental Pharmacology*.

[3] Zoń A., Bednarek I. (2023). Cisplatin in Ovarian Cancer Treatment—Known Limitations in Therapy Force New Solutions. *International Journal of Molecular Sciences*.

[4] Frezza M., Hindo S., Chen D. (2010). Novel Metals and Metal Complexes as Platforms for Cancer Therapy. *Current Pharmaceutical Design*.

[5] Ma L., Li L., Zhu G. (2022). Platinum-Containing Heterometallic Complexes in Cancer Therapy: Advances and Perspectives. *Inorganic Chemistry Frontiers*.

[6] Wheate N. J., Collins J. G. (2005). Multi-Nuclear Platinum Drugs: a New Paradigm in Chemotherapy. *Current Medicinal Chemistry: Anti-Cancer Agents*.

[7] Farrell N. P., De Almeida S. G., Skov K. A. (1988). Bis (Platinum) Complexes Containing Two Platinum Cis-Diammine Units. Synthesis and Initial DNA-Binding Studies. *Journal of the American Chemical Society*.

[8] Farrell N., Qu Y. (1989). Chemistry of Bis (Platinum) Complexes. Formation of Trans Derivatives from Tetraamine Complexes. *Inorganic Chemistry*.

[9] Farrell N., Qu Y., Hacker M. P. (1990). Cytotoxicity and Antitumor Activity of Bis (Platinum) Complexes. A Novel Class of Platinum Complexes Active in Cell Lines Resistant to Both Cisplatin and 1, 2-diaminocyclohexane Complexes. *Journal of Medicinal Chemistry*.

[10] Skov K. A., Adomat H., Farrell N. P., Matthews J. B. (1998). Assessment of Toxicity of Bis-Platinum Complexes in Hypoxic and Aerobic Cells. *Anti-Cancer Drug Design*.

[11] Broomhead J. A., Lynch M. J. (1995). The Synthesis and Characterization of Dinuclear Platinum Complexes Bridged by the 4, 4′-dipyrazolylmethane Ligand. *Inorganica Chimica Acta*.

[12] Broomhead J. A., Rendina L. M., Sterns M. (1992). Dinuclear Complexes of Platinum With the 4, 4′-dipyrazolylmethane Ligand. Synthesis, Characterization, and X-Ray Crystal Structure of. gamma.-bis (4, 4′-Dipyrazolylmethane-N, N′) Bis [dichloroplatinum (II)]-N, N-Dimethylformamide (1/2) and Related Complexes. *Inorganic Chemistry*.

[13] Zhang J., Wang L., Xing Z. (2010). Status of Bi-and Multi-Nuclear Platinum Anticancer Drug Development. *Anti-Cancer Agents in Medicinal Chemistry*.

[14] Colella G., Pennati M., Bearzatto A. (2001). Activity of a Trinuclear Platinum Complex in Human Ovarian Cancer Cell Lines Sensitive and Resistant to Cisplatin: Cytotoxicity and Induction and Gene-Specific Repair of DNA Lesions. *British Journal of Cancer*.

[15] Pratesi G., Perego P., Polizzi D. (1999). A Novel Charged Trinuclear Platinum Complex Effective against Cisplatin-Resistant Tumours: Hypersensitivity of P53-Mutant Human Tumour Xenografts. *British Journal of Cancer*.

[16] El-Gammal O. A., Mohamed F. S., Rezk G. N., El-Bindary A. A. (2021). Structural Characterization and Biological Activity of a New Metal Complexes Based of Schiff Base. *Journal of Molecular Liquids*.

[17] Johnson A., Qu Y., Van Houten B., Farrell N. (1992). B↑ Z DNA Conformational Changes Induced by a Family of Dinuclear Bis (Platinum) Complexes. *Nucleic Acids Research*.

[18] Zheng M., Liu S., Li J. (2014). Integrating Oxaliplatin With Highly Luminescent Carbon Dots: an Unprecedented Theranostic Agent for Personalized Medicine. *Advanced Materials*.

[19] Li J., Yap S. Q., Chin C. F. (2012). Platinum (IV) Prodrugs Entrapped Within Multiwalled Carbon Nanotubes: Selective Release by Chemical Reduction and Hydrophobicity Reversal. *Chemical Science*.

[20] Liang X.-J., Meng H., Wang Y. (2010). Metallofullerene Nanoparticles Circumvent Tumor Resistance to Cisplatin by Reactivating Endocytosis. *Proceedings of the National Academy of Sciences*.

[21] Slowing I., Trewyn B., Giri S., Lin V. (2007). Mesoporous Silica Nanoparticles for Drug Delivery and Biosensing Applications. *Advanced Functional Materials*.

[22] Cheng Z., Dai Y., Kang X. (2014). Gelatin-Encapsulated Iron Oxide Nanoparticles for Platinum (IV) Prodrug Delivery, Enzyme-Stimulated Release and MRI. *Biomaterials*.

[23] Dhar S., Daniel W. L., Giljohann D. A., Mirkin C. A., Lippard S. J. (2010). Polyvalent Oligonucleotide Gold Nanoparticle Conjugates as Delivery Vehicles for Platinum (IV) Warheads. *Journal of the American Chemical Society*.

[24] Min Y., Li J., Liu F., Yeow E. K., Xing B. (2014). Near‐infrared Light‐mediated Photoactivation of a Platinum Antitumor Prodrug and Simultaneous Cellular Apoptosis Imaging by Upconversion‐luminescent Nanoparticles. *Angewandte Chemie International Edition*.

[25] Johnstone T. C., Kulak N., Pridgen E. M., Farokhzad O. C., Langer R., Lippard S. J. (2013). Nanoparticle Encapsulation of Mitaplatin and the Effect Thereof on In Vivo Properties. *ACS Nano*.

[26] Zalba S., Garrido M. J. (2013). Liposomes, a Promising Strategy for Clinical Application of Platinum Derivatives. *Expert Opinion on Drug Delivery*.

[27] Ambegia E., Ansell S., Cullis P., Heyes J., Palmer L., MacLachlan I. (2005). Stabilized Plasmid–Lipid Particles Containing PEG-Diacylglycerols Exhibit Extended Circulation Lifetimes and Tumor Selective Gene Expression. *Biochimica et Biophysica Acta, Biomembranes*.

[28] Graf N., Bielenberg D. R., Kolishetti N. (2012). αVβ3 Integrin-Targeted PLGA-PEG Nanoparticles for Enhanced Anti-Tumor Efficacy of a Pt (IV) Prodrug. *ACS Nano*.

[29] Kapp T., Dullin A., Gust R. (2010). Platinum (II)− Dendrimer Conjugates: Synthesis and Investigations on Cytotoxicity, Cellular Distribution, Platinum Release, DNA, and Protein Binding. *Bioconjugate Chemistry*.

[30] Zhang Q., Kuang G., Zhang L., Zhu Y. (2023). Nanocarriers for Platinum Drug Delivery. *Biomedical Technology*.

[31] He X., Liu J., Song T. (2023). Effects of Water-Soluble Additive on the Release Profile and Pharmacodynamics of Triptorelin Loaded in PLGA Microspheres. *Drug Development and Industrial Pharmacy*.

[32] Park K., Skidmore S., Hadar J. (2019). Injectable, Long-Acting PLGA Formulations: Analyzing PLGA and Understanding Microparticle Formation. *Journal of Controlled Release*.

[33] Gonella A., Grizot S., Liu F., López Noriega A., Richard J. (2022). Long-Acting Injectable Formulation Technologies: Challenges and Opportunities for the Delivery of Fragile Molecules. *Expert Opinion on Drug Delivery*.

[34] Chaurasiya A., Patra P., Thathireddy P., Gorajiya A. (2021). PLGA-Based Micro-and Nano-Particles: From Lab to Market. *Micro-and Nanotechnologies-Based Product Development*.

[35] Mir M., Ahmed N., Rehman A. u. (2017). Recent Applications of PLGA Based Nanostructures in Drug Delivery. *Colloids and Surfaces B: Biointerfaces*.

[36] Ramu V., Gill M. R., Jarman P. J. (2015). A Cytostatic Ruthenium (II)–Platinum (II) Bis (Terpyridyl) Anticancer Complex That Blocks Entry into S Phase by Up‐regulating p27KIP1. *Chemistry--A European Journal*.

[37] Paziresh S., Babadi Aghakhanpour R., Rashidi M., Nabavizadeh S. M. (2018). Simple Tuning of the Luminescence Properties of the Double Rollover Cycloplatinated (II) Structure by Halide Ligands. *New Journal of Chemistry*.

[38] Morris G. M., Huey R., Lindstrom W. (2009). AutoDock4 and AutoDockTools4: Automated Docking With Selective Receptor Flexibility. *Journal of Computational Chemistry*.

[39] Hines D. J., Kaplan D. L. (2013). Poly (Lactic-co-glycolic) Acid− Controlled-Release Systems: Experimental and Modeling Insights. *Critical Reviews in Therapeutic Drug Carrier Systems*.

[40] Frisch A. (2009). *Gaussian 09W Reference*.

[41] Sousa da Silva A. W., Vranken W. F. (2012). ACPYPE-Antechamber Python Parser Interface. *BMC Research Notes*.

[42] Van Der Spoel D., Lindahl E., Hess B., Groenhof G., Mark A. E., Berendsen H. J. (2005). GROMACS: Fast, Flexible, and Free. *Journal of Computational Chemistry*.

[43] Evans D. J., Holian B. L. (1985). The Nose–Hoover Thermostat. *The Journal of Chemical Physics*.

[44] Parrinello M., Rahman A. (1981). Polymorphic Transitions in Single Crystals: A New Molecular Dynamics Method. *Journal of Applied Physics*.

[45] Essmann U., Perera L., Berkowitz M. L., Darden T., Lee H., Pedersen L. G. (1995). A Smooth Particle Mesh Ewald Method. *The Journal of Chemical Physics*.

[46] Van Gunsteren W. F., Berendsen H. J. (1988). A Leap-Frog Algorithm for Stochastic Dynamics. *Molecular Simulation*.

[47] Humphrey W., Dalke A., Schulten K. (1996). VMD: Visual Molecular Dynamics. *Journal of Molecular Graphics*.

[48] Liu Z.-C., Wang B.-D., Li B. (2010). Crystal Structures, DNA-Binding and Cytotoxic Activities Studies of Cu (II) Complexes With 2-Oxo-Quinoline-3-Carbaldehyde Schiff-Bases. *European Journal of Medicinal Chemistry*.

[49] Coban B., Sağlam C., Eser N., Babahan İ. (2017). Studying DNA Interactions of Ni (II) Complexes of Thiosemicarbazone Containing Vic-Dioxime Ligands. *Karaelmas Science & Engineering Journal*.

[50] Mahmood K., Akhter Z., Perveen F. (2023). Synthesis, DNA Binding and Biological Evaluation of Benzimidazole Schiff Base Ligands and Their Metal (II) Complexes. *RSC Advances*.

[51] Shabaninejad Z., Nikkhah M., Nabavizadeh S. M. (2023). DNA Binding Properties and Cytotoxic Effects of Two Double Rollover Cycloplatinated (II) Complexes on Cancer Cell Lines. *Journal of Inorganic Biochemistry*.

[52] Amini Khouzani A., Sohrabi N., Rasouli N., Eslami Moghadam M. (2018). A Spectroscopic Study on Calf Thymus DNA Binding Properties of Nickel (II) Complex With Imidazole Derivatives of 1, 10-phenanthroline Ligand. *Iranian chemical communication.*.

[53] Sohrabi N., Rasouli N., Kamkar M. (2014). Synthesis, Characterization and DNA Interaction Studies of (N, N′-bis (5-Phenylazosalicylaldehyde)-Ethylenediamine) Cobalt (II) Complex. *Bulletin of the Korean Chemical Society*.

[54] Selwin Joseyphus R., Sivasankaran Nair M. (2010). Synthesis, Characterization and Biological Studies of Some Co (II), Ni (II) and Cu (II) Complexes Derived from Indole-3-Carboxaldehyde and Glycylglycine as Schiff Base Ligand. *Arabian Journal of Chemistry*.

[55] Shabaninejad Z., Nikkhah M., Nabavizadeh M. (2021). Binding Studies of Two Double Rollovers Cycloplatinated Compounds and Bovine Serum Albumin by Fluorescence Spectroscopy. *Biomacromolecular Journal*.

[56] Bujalowski W. M., Jezewska M. J. (2012). Fluorescence Intensity, Anisotropy, and Transient Dynamic Quenching Stopped-Flow Kinetics. *Methods in Molecular Biology*.

[57] Askari A., Mokaberi P., Dareini M. (2021). Impact of Linker Histone in the Formation of Ambochlorin-Calf Thymus DNA Complex: Multi-Spectroscopic, Stopped-Flow, and Molecular Modeling Approaches. *Iranian Journal of Basic Medical Sciences*.

[58] Dolai M., Saha U., Kumar G. S., Ali M. (2018). Amidooxime‐Based Mononuclear Mn (II) Complexes: Synthesis, Characterization, and Studies on DNA Binding and Nuclease Activity. *ChemistrySelect*.

[59] Das S., Kumar G. S. (2008). Molecular Aspects on the Interaction of Phenosafranine to Deoxyribonucleic Acid: Model for Intercalative Drug–DNA Binding. *Journal of Molecular Structure*.

[60] Nehru S., Veeralakshmi S., Kalaiselvam S., Subin David S., Sandhya J., Arunachalam S. (2021). DNA Binding, Antibacterial, Hemolytic and Anticancer Studies of Some Fluorescent Emissive Surfactant-Ruthenium (II) Complexes. *Journal of Biomolecular Structure and Dynamics*.

[61] Alavianmehr M. M., Ashrafi A., Yousefi R. (2020). Anticancer Activity Assessment and DNA Binding Properties of Two Binuclear Platinum (II) Complexes Using Spectroscopic and Molecular Simulation Approaches. *Anti-Cancer Agents in Medicinal Chemistry*.

[62] Marković N., Zarić M., Živković M. D. (2019). Novel Platinum (II) Complexes Selectively Induced Apoptosis and Cell Cycle Arrest of Breast Cancer Cells In Vitro. *ChemistrySelect*.

[63] Senerovic L., Zivkovic M. D., Veselinovic A. (2015). Synthesis and Evaluation of Series of Diazine-Bridged Dinuclear Platinum (II) Complexes through In Vitro Toxicity and Molecular Modeling: Correlation between Structure and Activity of Pt (II) Complexes. *Journal of Medicinal Chemistry*.

[64] Romani A. M. (2022). Cisplatin in Cancer Treatment. *Biochemical Pharmacology*.

[65] Xiao X., Teng F., Shi C. (2022). Polymeric Nanoparticles—Promising Carriers for Cancer Therapy. *Frontiers in Bioengineering and Biotechnology*.

[66] Ding L., Agrawal P., Singh S. K., Chhonker Y. S., Sun J., Murry D. J. (2024). Polymer-Based Drug Delivery Systems for Cancer Therapeutics. *Polymers*.

[67] Wu J. (2021). The Enhanced Permeability and Retention (EPR) Effect: the Significance of the Concept and Methods to Enhance its Application. *Journal of Personalized Medicine*.

[68] Martins C., Sousa F., Araújo F., Sarmento B. (2018). Functionalizing PLGA and PLGA Derivatives for Drug Delivery and Tissue Regeneration Applications. *Advanced Healthcare Materials*.

[69] Zou L., Xiong S., Deng X. (2018). Preparation of Scutellarin Loaded TPGS Polymeric Micelles and Evaluation of its Pharmacokinetics and Pharmacodynamics Effects in Rats. *Acta Poloniae Pharmaceutica-Drug Research*.

[70] Toi H., Miura Y., Shibasaki S. (2012). Hepatic Sinusoidal Obstruction Associated With S-1 Plus Cisplatin Chemotherapy for Highly Advanced Gastric Cancer With Paraaortic Lymph Node Metastases: Report of a Case. *Clinical journal of gastroenterology*.

[71] Lees J. G., White D., Keating B. A. (2020). Oxaliplatin-Induced Haematological Toxicity and Splenomegaly in Mice. *PLoS One*.

