# Supplementary material for: In Silico, In Vitro, and In Vivo Investigations of Anticancer Properties of a Novel Platinum (II) Complex and Its PLGA Encapsulated Form
Source: Bioinorg Chem Appl. 2025 May 25;2025:2673015. doi: 10.1155/bca/2673015 (PMC12127131; doi:10.1155/bca/2673015)
Supplement: Supporting Information — Additional supporting information can be found online in the Supporting Information section. [file 2673015.f1.docx]

**Title:** **In Silico, In vitro, and In vivo Investigations of Anticancer properties of a Novel Platinum (II) Complex and its PLGA encapsulated form**

Zahra Shabaninejad ^1^, Mahdiyar Dehshiri ^1¥^, Sayed Mostafa Modarres Mousavi ^1¥^, Maryam Nikkhah ^1*^, Sadegh Shirian ^2, 3^, Sajad Moradi ^4^, S. Masoud Nabavizadeh ^5^

1. Department of Nanobiotechnology, Faculty of Biological Sciences, Tarbiat Modares University, P. O. Box: 14115-154, Tehran, Iran.
2. Department of Pathology, Faculty of Veterinary Medicine, Shahrekord University, Shahrekord, Iran.
3. Shiraz Molecular Pathology Research Center, Dr. Daneshbod Pathology Laboratory, Shiraz, Iran
4. Nano Drug Delivery Research Center, Health Technology Institute, Kermanshah University of Medical Sciences, P. O. Box: 7616913555 Kermanshah, Iran.
5. Department of Chemistry, College of Sciences, Shiraz University, P. O. Box: 84334 – 71946, Shiraz, Iran.

**Corresponding author:**

Maryam Nikkhah

[m_nikkhah@modares.ac.ir](mailto:m_nikkhah@modares.ac.ir)

Department of Nanobiotechnology, Faculty of Biological Sciences, Tarbiat Modares University, P. O. Box: 14115-154, Tehran, Iran.

¥ These authors are equally contributed to this work.


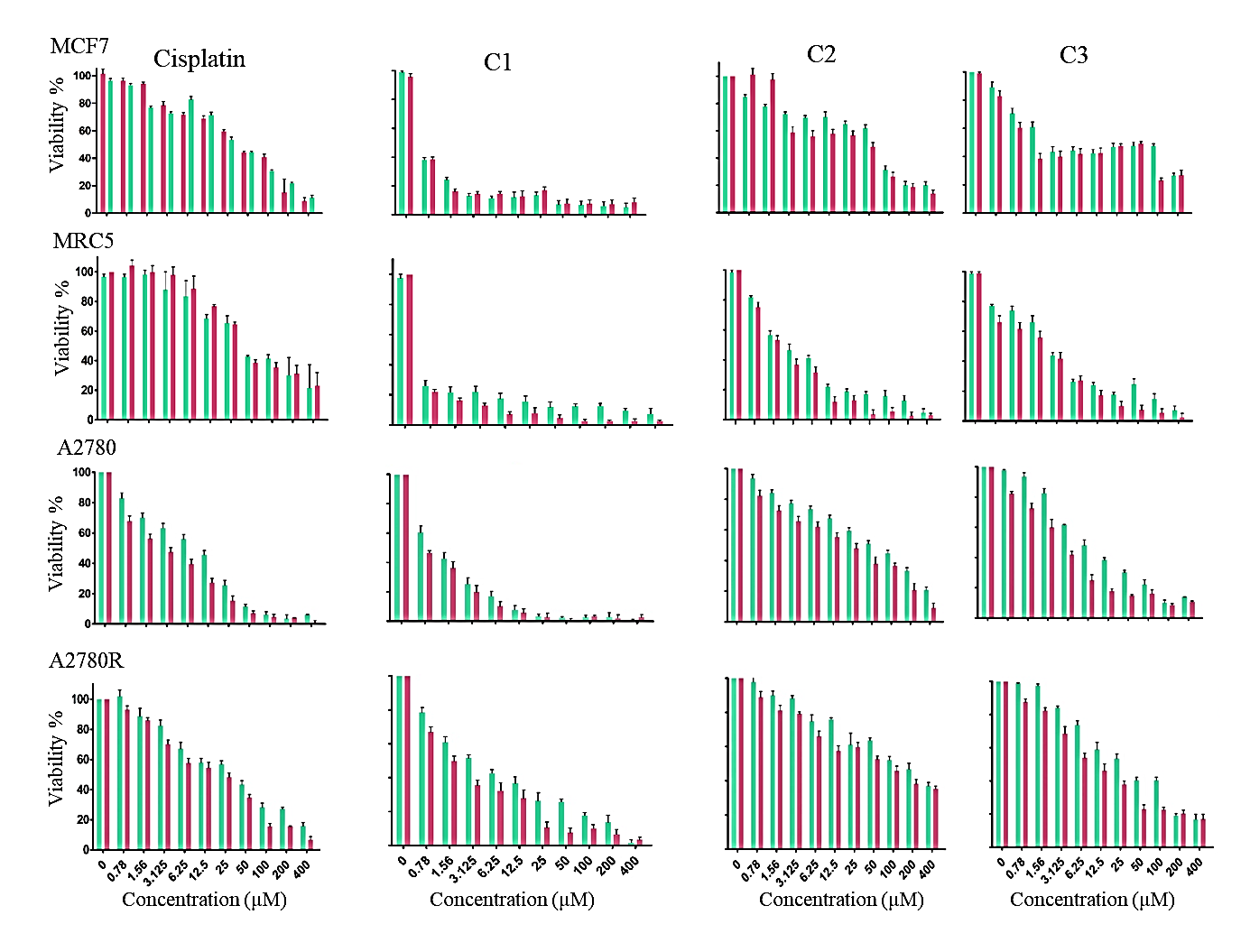


Figure S1 : Viability of MCF7, MRC5, A2780, and A2780R cells after treatment with different concentrations of cisplatin and C1. The cytotoxicity was measured by MTT assay after 24 h (green) and 48 h (purple) of treatments.


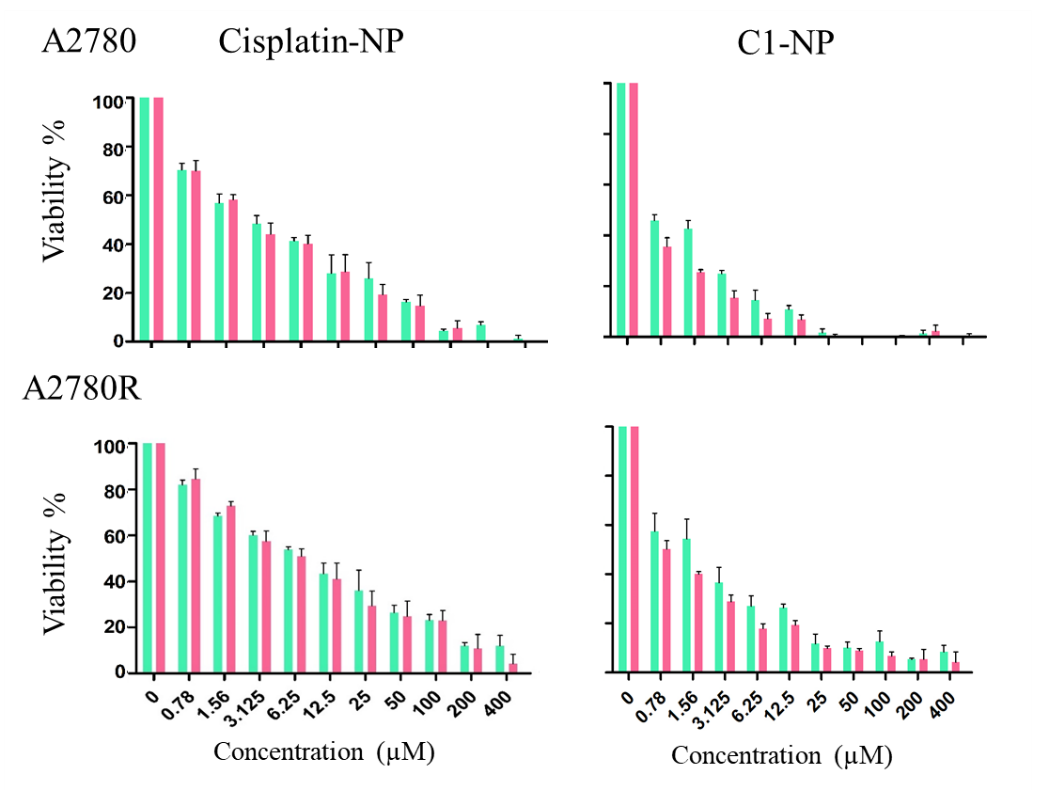


Figure S2: Cell viability of A2780 and A2780R cells after 24 h (green) and 48 h (purple) treatment by cisplatin-NP and C1-NP.


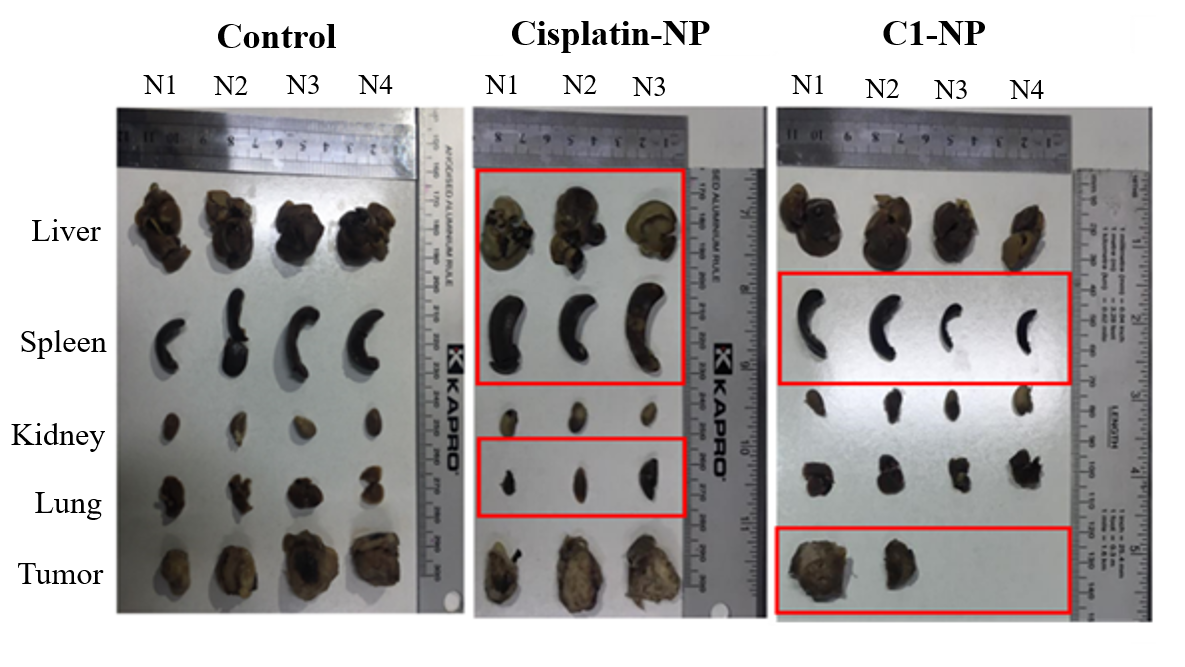


Figure S3. Morphology of organs in mice treated with C1-NP and cisplatin-NP compared to control.
